# Post-Partum Psychosis: Which Women Are at Highest Risk?

**DOI:** 10.1371/journal.pmed.1000027

**Published:** 2009-02-10

**Authors:** Phillipa J Hay

## Abstract

Phillipa Hay discusses the implications of a new study of psychotic illness and its risk factors up to 90 days postpartum in first-time Swedish mothers.

A range of psychological disorders occur in women in the post-partum period. These include “the blues”, which occurs in the first days after birth and which is very common and self-limiting; severe psychoses often associated with mania or bipolar illness, occurring in the first weeks after birth [1]; and mild to moderate depression, occurring weeks to months after birth.

## A New Study of Post-Partum Psychosis

In a new study published in *PLoS Medicine*, Unnur Valdimarsdóttir and colleagues have investigated the clinical incidence of psychotic illness and its risk factors up to 90 days post-partum in a large case register study of first-time Swedish mothers [2]. This study investigates an important problem, since the effects of maternal psychosis on attachment and infant care in the post-natal period can have lifelong adverse sequelae for mother and child [1]. The researchers argue correctly that whilst previous psychiatric illness is a well known risk factor for post-partum psychosis, it is not known how much other independent risk factors, such as obstetric complications, may be relevant. Only by studying first-time mothers with first psychotic episodes in the post-partum period can these independent risk factors begin to be elucidated.

Valdimarsdóttir and colleagues used a large (*n* = 745,596) national case register of hospital discharges over a 17-year period in Sweden. The study accorded with the STROBE guidelines for reporting cohort studies [3], and has a number of strengths. These include the large and representative sample, clear hypotheses, and appropriate statistics (estimating risk with proportional hazard regression models). There are two noted limitations. First is the lack of data on women with post-partum psychosis who are not hospitalised, although this would be rare. Second, this is an observational study—and while such studies are very good for raising further questions, they are less robust in drawing definitive explanations.

**Linked Research Article**

This Perspective discusses the following new study published in *PLoS Medicine*:

Valdimarsdóttir U, Hultman CM, Harlow B, Cnattingius S, Sparén P (2009) Psychotic illness in first-time mothers with no previous psychiatric hospitalizations: A population-based study. PLoS Med 6(2): e1000013. doi:10.1371/journal.pmed.1000013

Unnur Valdimarsdóttir and colleagues studied the risk factors for psychiatric illness following childbirth and found that, for women who had never previously been hospitalized for a psychiatric illness, the risk of mental illness was greatly increased following childbirth.

During the first 90 days post-partum, 892 women (1.2 per 1,000 births) were recorded as having been hospitalised due to psychoses. As expected, incidence rates for psychosis peaked in the first month after birth (285 of the 892 hospitalisations were in the first seven days, and 523 were in the first 14 days). About half of the hospitalisations (*n* = 436) were of women who had no previous psychiatric hospitalisation [2]. Specific to the first 90 days post-partum (but not later), higher maternal age was associated with increased risk of psychosis, and higher infant birth weight and maternal diabetes appeared protective. There was a different pattern of risk factors in the second 90-day period and in all women (i.e., including those with previous psychiatric hospitalisation). Overall, these findings suggest that there is a specific independent risk for psychosis in the early post-partum period.

## Does Estrogen Play a Role?

The authors correctly recognise that their findings cannot determine why the risk of psychosis is highest in the first month after birth. They postulate several explanations, favouring a biological vulnerability due to the profound hormonal fluctuations, particularly falls in estrogen levels, in this period. There is some empirical support for this hypothesis, to the degree that estrogen replacement has been proposed as a potential therapeutic agent in treating schizophrenia, with preliminary albeit inconclusive and mixed support from controlled trials [4,5]. However, as the authors report, a controlled trial found no support for the use of estrogen to prevent psychosis after childbirth [6]. Valdimarsdóttir and colleagues propose several hypotheses to explain the protective effects of diabetes and the greater risk in older mothers and those with infants of lower birth rate. They suggest that the risk factors may be mediated by greater estrogen depletion post-partum. However, the possibility that these may be proxy risk factors associated with some other more direct factor needs to be considered [7].

## First-Time Post-Partum Psychosis: A Distinct Condition?

Previous psychiatric illness remains an important risk factor for post-partum psychosis (as shown in the authors' previous work [8]), the treatment of post-partum psychosis is largely with anti-manic agents [1], and the future risk of bipolar disorder outside the post-partum period is high [9]. Nevertheless, there is some empirical support for regarding first-time post-partum psychosis as distinct from post-partum psychosis where there is a history of previous psychosis.

Robling and colleagues reported less likelihood of further psychotic episodes where the psychosis arose after the first childbirth, and particularly in the first post-partum month [9]. In a case record study in North West Wales, Tschinkel and colleagues compared post-partum psychosis during two time periods, 1875–1924 and 1994–2005 [10]. They found that rates of “first-onset” post-partum psychosis have fallen since the first period, while rates associated with previous psychiatric disorder remain the same. Why this should be so is unclear, but factors outside biological hormonal fluctuations must be considered—because hormonal fluctuations are unlikely to have changed to the degree that social, economic, or wider factors related to health care have done in the twentieth century.

## Future Research Directions

The new study raises important questions for future research [2]. To determine pathways to psychosis, and which risk factors are proxy, overlapping, truly independent, mediating, or moderating [7], there need to be targeted empirical prospective studies of new mothers at high risk and/or identified early in the post-natal psychosis. Such prospective studies would also enable delineation of the temporal sequence of putative biological but also social and demographic variables.

It is unlikely that there will be any single explanation, and that biological factors will be independent of others. For example, there are a wide range of biological, social, and psychological causes of low birth weight including caffeine use [11], maternal age, maternal depression [12], medical factors, and economic variables [13], many of which are not independent. If we can elucidate the risk factors for first-time de novo post-partum psychosis, a rare but devastating psychiatric illness, we can then test potential new treatments to redress mediating and moderating factors and thus help reduce the impact and burden.

## References

[pmed-1000027-b001] Sit D, Rothschild AJ, Wisner KL (2006). A review of postpartum psychosis. J Womens Health (Larchmt).

[pmed-1000027-b002] Valdimarsdóttir U, Hultman CM, Harlow B, Cnattingius S, Sparén P (2009). Psychotic illness in first-time mothers with no previous psychiatric hospitalizations: A population-based study. PLoS Med.

[pmed-1000027-b003] von Elm E, Altman DG, Egger M, Pocock SJ, Gøtzsche PC (2007). The Strengthening the Reporting of Observational Studies in Epidemiology (STROBE) Statement: Guidelines for reporting observational studies. PLoS Med.

[pmed-1000027-b004] Kulkarni J, de Castella A, Fitzgerald PB, Gurvich CT, Bailey M (2008). Estrogen in severe mental illness: A potential new treatment approach. Arch Gen Psychiatry.

[pmed-1000027-b005] Chua WL, de Izquierdo SA, Kulkarni J, Mortimer A (2005). Estrogen for schizophrenia. Cochrane Database Syst Rev.

[pmed-1000027-b006] Kumar C, McIvor RJ, Davies T, Brown N, Papadopoulos A (2003). Estrogen administration does not reduce the rate of recurrence of affective psychosis after childbirth. J Clin Psychiatry.

[pmed-1000027-b007] Kraemer HC, Stice E, Kazdin A, Offord D, Kupfer D (2001). How do risk factors work together? Mediators, moderators, and independent, overlapping, and proxy risk factors. Am J Psychiatry.

[pmed-1000027-b008] Harlow BL, Vitonis AF, Sparen P, Cnattingius S, Joffe H (2007). Incidence of hospitalization for postpartum psychotic and bipolar episodes in women with and without prior prepregnancy or prenatal psychiatric hospitalizations. Arch Gen Psychiatry.

[pmed-1000027-b009] Robling SA, Paykel ES, Dunn VJ, Abbott R, Katona C (2000). Long-term outcome of severe puerperal psychiatric illness: A 23 year follow-up study. Psychol Med.

[pmed-1000027-b010] Tschinkel S, Harris M, Le Noury J, Healy D (2007). Postpartum psychosis: Two cohorts compared, 1875–1924 and 1994–2005. Psychol Med.

[pmed-1000027-b011] CARE Study Group (2008). Maternal caffeine intake during pregnancy and risk of fetal growth restriction: A large prospective observational study. BMJ.

[pmed-1000027-b012] Kabir K, Sheeder J, Stevens-Simon C (2008). Depression, weight gain, and low birth weight adolescent delivery: Do somatic symptoms strengthen or weaken the relationship. J Pediatr Adolesc Gynecol.

[pmed-1000027-b013] Hunter KG, Taslimi MM (2008). Variation in infant birth weight: Socioeconomic factors versus medical conditions. J Health Hum Serv Adm.

